# Transformations in the Photogrammetric Co-Processing of Thermal Infrared Images and RGB Images

**DOI:** 10.3390/s21155061

**Published:** 2021-07-26

**Authors:** Adam Dlesk, Karel Vach, Karel Pavelka

**Affiliations:** 1 Department of Geomatics, Faculty of Civil Engineering, Czech Technical University in Prague, 16629 Prague, Czech Republic; pavelka@fsv.cvut.cz; 2 EuroGV, Spol. s. r. o., 11000 Prague, Czech Republic; vach@eurogv.cz

**Keywords:** photogrammetry, thermal infrared image, thermal camera calibration, relative pose estimation, close-range photogrammetry

## Abstract

The photogrammetric processing of thermal infrared (TIR) images deals with several difficulties. TIR images ordinarily have low-resolution and the contrast of the images is very low. These factors strongly complicate the photogrammetric processing, especially when a modern structure from motion method is used. These factors can be avoided by a certain co-processing method of TIR and RGB images. Two of the solutions of co-processing were suggested by the authors and are presented in this article. Each solution requires a different type of transformation–plane transformation and spatial transformation. Both types of transformations are discussed in this paper. On the experiments that were performed, there are presented requirements, advantages, disadvantages, and results of the transformations. Both methods are evaluated mainly in terms of accuracy. The transformations are presented on suggested methods, but they can be easily applied to different kinds of methods of co-processing of TIR and RGB images.

## 1. Introduction

Photogrammetric processing of close-range thermal infrared (TIR) images deals with several challenging difficulties. The resolution of TIR images is much lower compared to the resolution of images captured by common digital RGB cameras. The field of view (FOV) of thermal infrared cameras is generally much narrower. When the task is to inspect a large object by a TIR camera, usually many images must be captured. The high amount of images greatly complicates the thermal inspection of the object. Easing the inspection would include creating the super-resolution image [1] or creating, e.g., point cloud, a 3D textured model, or the orthophoto generated by photogrammetric processing using the structure from motion (SfM) method.

**Figure 1:**
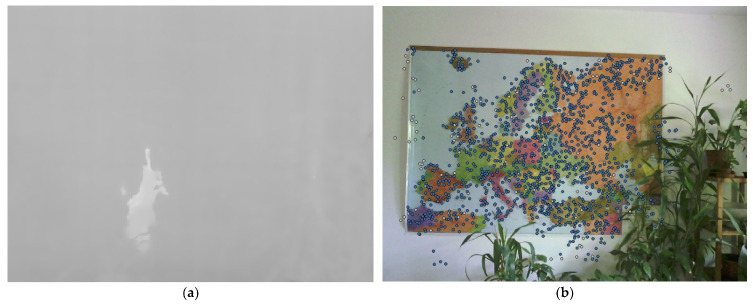
TIR and RGB image of the same scene: (**a**) feature matching algorithm failed to find any key point or tie point; (**b**) feature matching algorithm found sufficient amount of tie points (blue dots).

The SfM processing of TIR images is complicated by several factors. In addition to aforementioned issue of low-resolution of TIR images, the contrast of the TIR images depends on the variance of the thermal radiation of the scene. Where the scene is in a more or less similar temperature environment, e.g., interior of the room, building façade, etc., the TIR image can have very low contrast and the image lacks features. This is a crucial complication for processing using the structure from motion (SfM) method. The algorithms of SfM are unable to find a sufficient amount of key and tie points (see Figure 1a), which significantly complicates the calculation of relative orientation, reduces the accuracy, or even makes the computation impossible.

Another factor that makes photogrammetric processing of TIR images challenging is that the conventional way of photogrammetric targeting is not usable, because the targets are not detectable on TIR images (ordinarily, there is no difference in thermal radiation on targets). These factors complicate the scaling or georeferencing of the results of the SfM processing.

All mentioned factors must be taken into account. To eliminate these negative factors, several methods of photogrammetric processing of TIR images and RGB images together were presented. The motivation of the co-processing is presented in the example (Figure 1). In this example, it is apparent that the TIR image lacks any features so the SfM algorithm for feature matching failed to find any tie points. On the other hand, the RGB image of the same scene has identifiable features; the algorithm was successful in feature matching, resulting in a sufficient number of tie points on the RGB image. The main idea of the co-processing is to use the features with the quality of the RGB image and the thermal information from the TIR image.

Photogrammetric co-processing of TIR and RGB images was presented in several prior research contributions. The reported methods can be divided into two categories. The first category of methods does not require capturing the corresponding RGB image to every TIR image in the same configuration during the capturing. The scene can be captured by a TIR camera and RGB camera separately, even at different times. The second category requires a fixed system of TIR and RGB cameras and for every TIR image, an RGB image must be captured. In both categories, the results of the photogrammetric processing of TIR images are 2D and 3D models (orthophotos, point clouds, and textured models), which are augmented by information which is derived from the original TIR image. These results can help understand better the thermal behavior of objects of interest or localize the thermal anomalies in the reference coordinate system and determine their scope. This could be efficiently used in the different areas of research.

The following methods belong to the first category. A method presented by [2] consists of the processing of RGB and TIR images separately. Based on the images, the point clouds were obtained from the SfM method. Both of the generated point clouds were co-registered based on known position of the images gathered from GNSS. Then, for following 3D model generation, the author used the geometric quality from the processing of RGB images, and the texture was applied from TIR images. A similar idea was presented by [3]. RGB images were processed by the SfM method and the digital model of surface was generated. Then the group of TIR images was processed by the SfM method as well. When the final orthophoto of the scene was generated, the TIR images were applied on generated DSM (created from RGB images). Reference [4] presented a method where the scene was captured by TIR images and RGB images separately. Each group was then processed separately by the SfM method and the point clouds from each group were generated. Then both point clouds were co-registered by the ICP algorithm and the point cloud from RGB images was used for DSM generation. The texture from TIR images was then applied to the DSM and the result was the 3D textured model with the thermal infrared information. Reference [5] presented a method of generating a point cloud from TIR images captured by a TIR camera and a point cloud from RGB images captured by a DSLR camera. Images were processed separately using the SfM method; from both groups of images, colored point clouds were generated. Then the point clouds were co-registered using the ICP method. Then every point of the RGB point cloud was augmented by thermal information, which was derived from the nearest point from the TIR point cloud. The nearest points were found by the nearest neighbor search function. The result was point cloud augmented by thermal information.

Several methods belong to the category that requires a fixed system of cameras and capturing corresponding images. Developing methods in this category is supported by the fact that modern handheld industrial thermal infrared cameras (which are suited for close-range inspection of the objects) are equipped with both thermal infrared sensors and digital RGB sensors. The cameras are usually lightweight, easily operable, and have the potential of being used for photogrammetric purposes. Those cameras are not the only option—another way could be to create one’s own fixed system of TIR camera and RGB camera. A paper by [6] belongs in this category of methods. Different co-registration methods were presented, including methods of co-registering corresponding images based on features or based on 2D line segments, methods of point cloud co-registering (each point cloud was generated from TIR and RGB images separately), by the iterative closest point (ICP) algorithm, and by corresponding segmented planes.By [7], a PANTIR dual camera setup that consisted of a panchromatic and TIR camera carried on the helicopter was described. Both cameras were almost parallel to each other. The registration between corresponding images was carried out using plane transformation. The author mentions the fact that, in the case of a non-parallel optical axis, the images must then be taken at constant altitude. Moreover, before the fusion of the images, both images must be undistorted. Then the corresponding RGB and TIR images are fused to the PANTIR images and the process of photogrammetric processing using SfM continues. A method presented by [8] was using a modern industrial thermal infrared camera FLIR T1030sc equipped with a TIR sensor and an RGB sensor in a fixed configuration was presented. For every TIR image, an RGB image was captured. Then both sensors were geometrically calibrated in order to undistort the images. The transformation key of the projective transformation was computed and was used for all corresponding image pairs. Then the scene was captured by a FLIR T1030sc camera and by a DSLR RGB camera. The images captured by the DSLR camera were processed using the SfM method together with RGB images captured by a FLIR T1030sc camera. The result of the processing was a DSM. Then the RGB images captured by the FLIR T1030sc camera were replaced by the corresponding undistorted and geometrically corrected TIR images. Only TIR images were used for the final generation of a texture for the 3D model. The final result of the process was an orthophoto augmented by thermal information. By [9], a method of photogrammetric co-processing of TIR images and RGB images was presented. The corresponding images (RGB and TIR images) were co-registered either by plane transformation (concretely by affine transformation) or by 3D spatial transformation. Overall, the important topic of this category of papers is the transformation between the images captured by cameras in a fixed configuration. One approach is to use 2D plane transformation and the second approach is to use 3D spatial transformation. The plane transformation was used by [7], concretely [8] used projective transformation and [9] used affine transformation. Spatial transformation was used by [9] as well. Previously, authors of this article suggested two methods of co-processing the TIR and RGB images, which were captured by the fixed camera system [10]. Both methods use SfM processing. The first method was called Sharpening (according to the pan-sharpening method); for this method, plane transformation was designed. The result of this method is mainly augmented orthophoto. The second method is called Reprojection because it vastly depends on the accurate reprojection process. Our method uses spatial transformation and the result of the method is a point cloud.

The focus of this article is the transformation between the images captured by the fixed camera system. This article describes the process of the transformations, different requirements of transformation, expected accuracy of transformation processes, results, and potential usability for TIR and RGB image co-processing. The paper discusses the plane transformation, which was designed for the Sharpening method, and spatial transformation, which was designed for the Reprojection method. Using the plane transformation requires finding a universal transformation key between the TIR image and the corresponding RGB image. In this paper, an experiment examines if the transformation key is dependent on the scene, and how the scene influences the accuracy of the transformation). In the literature, the images are captured in most of the cases from larger distance, oftentimes from UAV, e.g., [2,3,4,6,7,9,11,12,13]. However, interest from the authors of this article involves a close-range application. Thus, capturing for the experiments was done at close range (from 2 to 11 m, approximately). In those distances, the scene changed a lot during the capturing. Spatial transformation required accurate relative poses and relative orientation between the TIR and RGB cameras mounted on a fixed rig and determination of the internal orientation of the cameras. This paper presents a process of geometric calibration of the cameras, determination of interior orientation parameters, and determination of the relative orientation and pose. Then the calculated parameters were tested on an experiment and the accuracy of the spatial transformation was evaluated.

Both suggested methods (Sharpening and Reprojection), which are, for context, briefly presented in this article as well, are in development, and the results of this article are needed for final implementation. Even though the transformations are presented on the two suggested methods, the knowledge and the results of the experiments can be used in a variety of methods of photogrammetric co-processing of TIR and RGB images.

### 1.1. Suggested Methods

Two methods of photogrammetric co-processing of TIR images, together with corresponding RGB images, were suggested by the authors of this article. These methods are called Sharpening and Reprojection. In both suggested methods, there is the presumption of having a fixed relative pose between the TIR camera and RGB camera, and for every TIR image having captured a corresponding RGB image. Both methods were designed for photogrammetric processing by the structure from motion method. Both methods were previously presented by the authors of this article [10]. The methods are still in development and both rely on correct transformation processes. Results of this article are needed to finish the development and implementation.

#### 1.1.1. Sharpening and Plane Transformation

**Figure 2:**
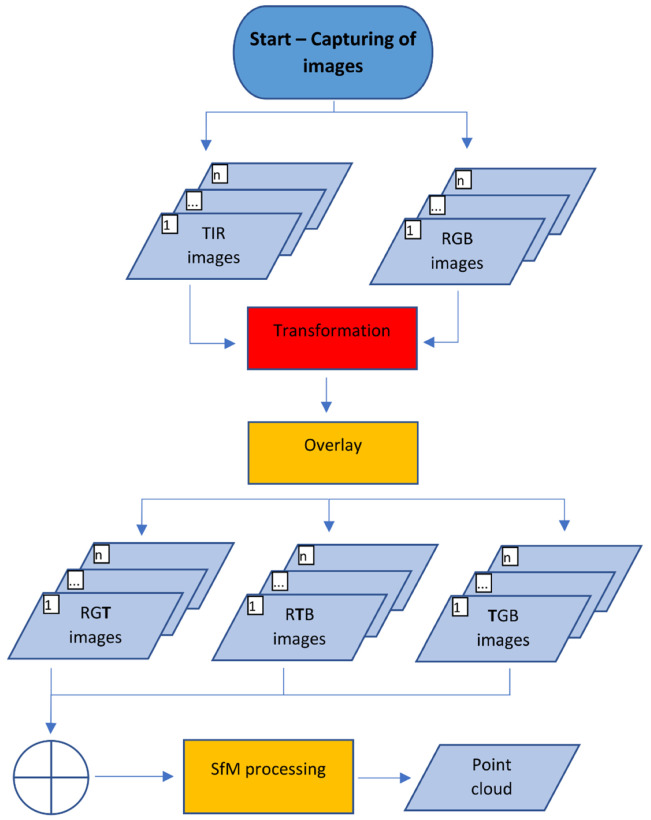
Diagram of the Sharpening method.

For the Sharpening method (see Figure 2), during the capture of the TIR images, for every TIR image, a corresponding RGB image was captured. The capturing scenario is the same as suggested for processing by the SfM method. Before the processing, the TIR image was transformed by a plane transformation to the RGB image to create an overlay of the images. The same transformation key was then used for all other image pairs. Presumptions for sufficient plane transformations are that both cameras are placed close next to each other, both cameras have more or less parallel optical axes, and images of the captured cameras are undistorted. To create the overlay, one of the channels of the RGB image was skipped and replaced by values from the thermal infrared image, which were recalculated by a function to 8-bit values (0–255) or 16-bit values. By this process (in case of skipping the blue channel of the RGB image), a sharpened false-colored RGT image was created, where visible information from the RGB camera (channels RG) and thermal infrared information from the TIR image (channel T) was partly stored. An RTB or TGB combination could be analogously created, or even 4-channel RGBT images. This process was carried out for every image pair of TIR and RGB images.

The group of sharpened images was then processed by SfM using the standard workflow, the relative and absolute orientation was calculated and then a point cloud, a 3D model, and an orthophoto were created. The result of this method is orthophoto augmented by thermal infrared information. The orthophoto has false colors (e.g., RGT colors). For every pixel of the orthophoto, the thermal information was calculated back from the T value in the pixel using the inverse previously used function. The thermal information was then stored in the pixel.

For the transformation in the Sharpening method, a plane transformation [14] was designed. Concretely, affine transformation and projective transformation were considered. As stated below, both transformations were tested and gave more or less similar results. Testing of the affine transformation provided better results; that is why it is presented and used for the following experiments.

One way how to determine the coordinate transformation between pixels in an RGB image $P_{RGB}$ and pixels in a TIR image $P_{TIR}$ is the plane transformation. In this article, affine transformation is used; see Equation (1).

(1) $$P_{TIR}=\left[\begin{array}{cc} m_{X}\;\cos\alpha & -m_{Y}\;\sin\left(\alpha +\beta \right)\\ m_{X}\;\sin\alpha & m_{Y}\;\cos\left(\alpha +\beta \right) \end{array}\right]P_{RGB}+T$$

For purposes of the Sharpening method, on the selected image pairs, it is necessary to find a transformation key, respectively, parameters of affine transformation rotation angle α, shearing angle β, scaling factor $m_{X}$, $m_{Y}$ and translation T between the images. The same transformation key is then applied to the rest of the image pairs. Presumptions for having sufficient results of the transformation include having both cameras next to each other and having their optical axes parallel, as much as is possible. The accuracy of the transformation is also dependent on the scene, on the images, and the distance to the scene. The testing of the influence of the distance is carried out in this paper. Then the results and sufficiency of affine transformation are discussed.

#### 1.1.2. Reprojection and Spatial Transformation

**Figure 3:**
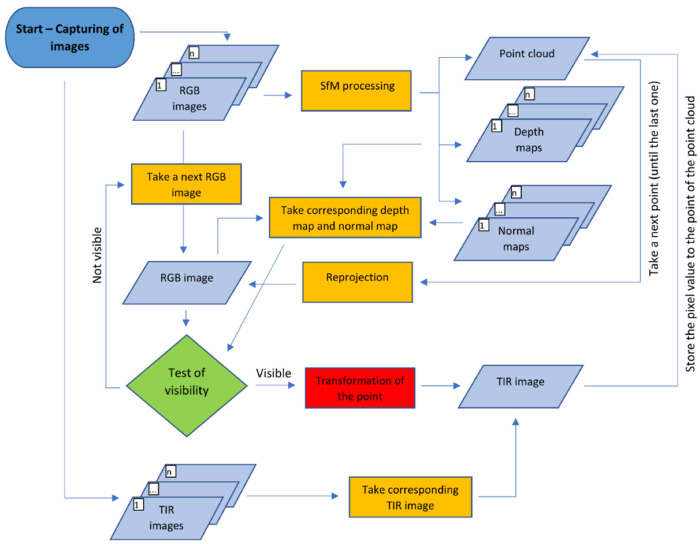
Diagram of the Reprojection method.

In the Reprojection method (see diagram Figure 3), there is the presumption that, during the capturing scenario, the relative pose of the TIR camera and RGB camera is fixed and a corresponding RGB image is captured for every TIR image. During the capture, the optical state of the cameras must not be changed. Then, only RGB images are photogrammetrically processed by the SfM method and the point cloud is generated. Then every point of the point cloud is reprojected to the automatically, semi-automatically, or manually chosen set of RGB images, where the point of the point cloud is potentially visible. Then it is necessary to carry out “test of visibility” for every image, to check if the point of the point cloud is truly visible on the RGB image. It is possible to carry out the visibility test, for example, using depth and the normal map of the tested RGB image from the processing, using the SfM method. The depth and normal map are rendered, based on the 3D model, which is generated based on the dense point cloud from RGB images. If the test of visibility, based on the corresponding depth map and normal map, proves that the point is visible on the RGB image, the reprojected point on the RGB image is transformed by a spatial transformation to the TIR image. The thermal infrared information can be read from the pixel on the TIR image (where the point was transformed) and the thermal infrared information can be added to the point in the point cloud. Then it is possible to create a colored point cloud with thermal infrared information.

As the transformation for the Reprojection method, spatial transformation was designed [14]. Having a point ***P****_w_* in world coordinates, and knowing the parameters of the exterior orientation of the RGB image, i.e., rotation matrix ***R****_RGB_* consisting of rotation angles *ω*, *φ*, *κ*, and translation ***T**_RGB_* position of the camera in world coordinates, consisting of *X_RGB_*, *Y_RGB_*, *Z_RGB_*, it is possible to transform the point and get the coordinates of the point in the RGB camera coordinate system $P_{C_{\mathrm{RGB}}}$.

(2) $$R=R_{\omega }R_{\varphi }R_{\kappa },\;\;\;T_{RGB}=\left[\begin{array}{c} X_{RGB}\\ Y_{RGB}\\ Z_{RGB} \end{array}\right]$$

(3) $$P_{C_{RGB}}=R_{RGB}^{-1}(P_{w}-T_{RGB})$$

Knowing the rotation matrix $R_{RGB\to TIR}$ and translation $T_{RGB\to TIR}$ directly between the RGB camera coordinate system and the TIR camera coordinate system, it is possible to transform the point $P_{C_{\mathrm{RGB}}}$ to the TIR camera coordinate system $P_{C_{\mathrm{TIR}}}$.

(4) $$R_{RGB\to TIR}=R_{TIR}^{-1}R_{RGB}$$

(5) $$T_{RGB\to TIR}=R_{RGB}^{-1}(T_{TIR}-T_{RGB})$$

(6) $$P_{C_{TIR}}=R_{RGB\to TIR}(P_{C_{RGB}}-T_{RGB\to TIR})$$

Then, from the TIR camera coordinate system, knowing the calibration matrix of the TIR camera ***K**_TIR_*, it is possible to reproject the point $P_{C_{\mathrm{TIR}}}$ to the thermal infrared image and get the position on the image ***P****_TIR_*.

(7) $$K_{RGB}=\left[\begin{array}{ccc} c & s & p_{x}+\Delta x\\ 0 & c\left(1+m\right) & p_{y}+\Delta y\\ 0 & 0 & 1 \end{array}\right]$$

(8) $$P_{TIR}=K_{TIR}P_{C_{TIR}}$$

Calibration matrix ***K**_TIR_* consists of camera constant *c*, *p_x_* and *p_y_* are the coordinates of the principal point of the camera, *m* is the scale difference, and *s* is sheer. Parameters Δx and Δy are the calculated values of radial distortion for the concrete pixel.

This process requires the determination of the rotation matrix $R_{RGB\to TIR}$ between the RGB and TIR camera coordinate systems, the translation $T_{RGB\to TIR}$, and calibration matrix $K_{TIR}$. The rotation matrix $R_{RGB\to TIR}$ is calculated by Equation (4) and can then be decomposed to differences in rotation for each axis between the camera coordinate systems Δω, Δφ, and Δκ. The translation vector consists of ΔX, ΔY, and ΔZ. The considered parameters of the calibration matrix are *c*, *p_x_*, *p_y_*, and coefficients of radial distortion *k_1_* and *k_2_*. The determination of those parameters is problematic because it requires a process of geometric calibration of both the TIR camera and RGB camera, and then processing of TIR and RGB images together with highly accurate determination of the exterior orientation of the image pairs. The determination of the relative pose and process of geometric calibration is discussed in this paper in the following experiments.

### 1.2. Handheld Thermal Infrared Cameras

For the capture of close-range thermal infrared images in common industrial applications, it is suitable to use handheld TIR cameras. The handheld thermal infrared cameras are usually light and durable. Modern cameras are also equipped with digital RGB cameras. It is possible to use the cameras separately or for every captured TIR image, the corresponding RGB image. The main reason as to why the TIR cameras are equipped with RGB cameras is to achieve better orientation or better visual impressions during the thermal infrared inspection. The software for inspection of thermal infrared images usually includes the functions of overlaying the TIR images and corresponding RGB images. The software also has functions of gathering the edges from digital RGB images, projecting the edges to the TIR images for better orientation in TIR images. These features help to better understand the results of TIR imaging and lead to better inspections overall. However, the fact that handheld TIR cameras are equipped with RGB sensors is applicable for purposes of photogrammetry, which helps the suggested methods by authors who require a fixed system of RGB and TIR cameras.

According to Table 1, the resolution of TIR images on handheld cameras starts from 320 × 240 pixels to 640 × 480 pixels. The resolution of digital cameras is according to the public technical specifications (5 megapixels). The 5 MP resolution of RGB cameras is a standard among handheld TIR cameras, but it is below standard compared to the professional digital cameras, which are commonly used in photogrammetry. Nevertheless, the 5 MP resolution of the digital camera promises to achieve better and more accurate results of photogrammetric processing.

The FLIR E95 thermal infrared camera, which was used for the experiments in this paper, is equipped with an uncooled microbolometer thermal sensor with dimension 7.888 × 5.916 mm and resolution 464 × 348 pix. The pixel pitch of the sensor was 17 μm. The resolution of the TIR image, compared to the other presented handheld TIR cameras, is in the mid-range category. FLIR E95 is possibly equipped with different lenses. The 29 mm lens has the longest focal length and the narrowest field of view (FOV) 14° × 10°. The 10 mm lens has the widest FOV 42° × 32°, but the FOV is still narrow compared to the common digital cameras. This factor also complicates the capturing of the TIR images for photogrammetric processing. Only the 10 mm lens was used for the experiments of this paper.

The lenses of the TIR camera of FLIR E95 are focusable with a focus ring. The digital camera of FLIR E95 has a resolution of 5MP, specifically 2592 × 1944 pixels. The focal length of the lens of the RGB camera is 3.29 mm and the field of view is 53° × 41°, which is wider than the FOV of the 10 mm lens on the TIR camera. The focus of the lens of the digital RGB camera is fixed, according to the manual [15]. A summary of the parameters of the TIR camera and RGB camera on FLIR E95 is presented in Table 2.

## 2. Affine Transformation for Sharpening Method

Testing—using the plane transformation for the Sharpening method—was conducted. For the testing, the FLIR E95 camera was used. FLIR E95 is a TIR camera equipped with a digital RGB camera, and for every TIR image, it is possible to capture a corresponding RGB image. Both cameras are naturally in a fixed position to each other. The goal of the testing was to examine if there was a universal transformation key that could be applied to all image-pairs of the dataset or if there was a dependency on the distance, which could complicate the process of transformation in the Sharpening method, and how high the influence of the different distance was. Before the testing, some of the presumptions were done. The first presumption was that both cameras are close to each other (the RGB camera is approximately 3 cm above the TIR camera). Both cameras have, by eye, parallel optical axis. Both cameras have fixed focusing during the capturing. Moreover, the images must be undistorted before the transformation process. Pilot projects of geometric calibration show that both cameras are practically undistorted. The process of undistortion is probably carried out by internal processes of the FLIR E95 camera because even the camera itself has overlay functions. The radial distortion of the FLIR E95 camera is described more in Section 3.1.

**Figure 4:**
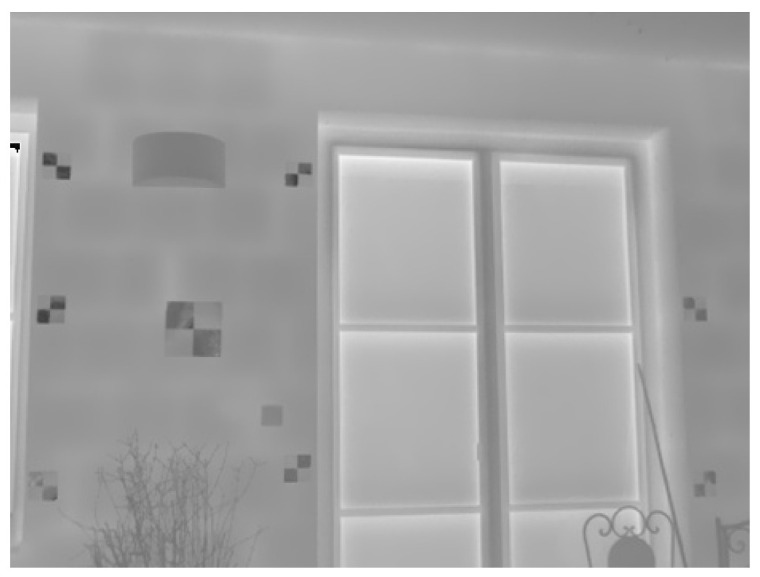
Image captured from 4 m distance. In the image are recognizable cross aluminum paper targets.

As a test object, a façade of a family house with a plane structure was chosen. On the façade, cross aluminum paper photogrammetric targets were placed and equally distributed. According to the previously mentioned literature, classic photogrammetric targets are not often used for capturing TIR images. To achieve visibility of the targets, instead of a color difference, there must be a difference in thermal radiation, which is created by using a different emissivity of the two used materials. Instead of a black color, aluminum material was used to achieve dark color on the thermal image. Aluminum foil is a material with a low emissivity (emissivity ε = 0.04 [17]), so instead of emitting the thermal radiation itself, aluminum reflects the thermal radiation of the surrounding ambiance. As a white color, a paper was used on the target, which has high emissivity and mainly emits thermal radiation (ε = 0.93 [17]). In praxis, having a cross aluminum paper target placed on the façade, the center of the target is visible on the TIR image because the aluminum part reflects thermal radiation of the cold of the sky (dark color in our case), and paper (bright color) emits thermal radiation—significantly warmer itself (see Figure 4). Similar targets were used in [5]. However, in some cases, even black and white printed photogrammetric targets could be visible on TIR images. While the paper has high emissivity, printed ink has lower emissivity (ε = 0.83 [18]). The same effect is achieved by having aluminum paper cross targets; it is possible to achieve by ink-paper targets, but with significantly lower emissivity difference.

After the photogrammetric targeting, 10 stations (from 2 m from the façade to 11 m) were made. Those short distances were chosen because our goal was to focus on close-range TIR photogrammetry. From each station, the TIR image and the corresponding RGB image were captured. During imaging, the focus of the TIR camera was fixed to the 7 m distance. The RGB camera was fixed focus. All images were captured perpendicular to the façade, so all of the photogrammetric targets in the images were at a more or less same distance to the image. An image example is presented in Figure 4. Approximate sample distances of the captured image pairs are presented in Table 3.

**Figure 5:**
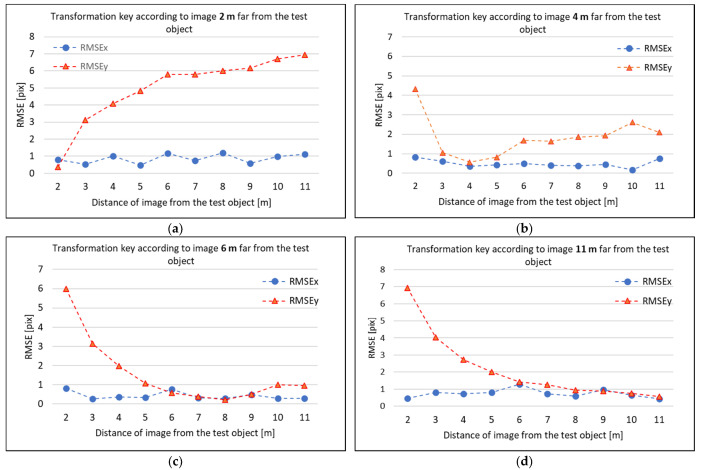
RMSEx and RMSEy of residuals on the TIR image transformed to the RGB image using affine transformation: (**a**) transformation key computed on 2 m image-pair; (**b**) transformation key computed on 4 m image-pair; (**c**) transformation key computed on 6 m image-pair; (**d**) transformation key computed on 11 m image-pair.

The affine transformation was chosen. The projective transformation was tested and the results were more or less the same or slightly worse. Thus, the affine transformation was chosen. For the affine transformation, a transformation key between the TIR image and RGB image of one image pair was calculated, i.e., tested to see if the transformation key calculated on one chosen pair was universal and sufficient enough for all other image pairs of the dataset. At first, the transformation key calculated on the image pair captured from the station 2 m from the test object was used. The transformation itself resulted in 0.4 pixel RMS. Then the same transformation key was used for the other pairs captured from other stations (3–11 m). For all distances, the transformed TIR images were overlaid on the corresponding RGB images and the residuals were measured on the photogrammetric targets on TIR images. From the residuals, RMSE for each axis on the TIR images were calculated. The X-axis of the images went from the image center to the right and Y-axis from the center image perpendicularly downwards. The resulting RMSEs are presented in Figure 5a, and are presented separately for each axis (RMSEx, RMSEy). The same procedure was repeated again, but the transformation key was computed in image pairs captured from station 4 m from the test object (Figure 5b)), then in image pairs, captured 6 m from the test object (Figure 5c)) and 11 m (Figure 5d)). All of the presented RMSE pixel values are related to the TIR image pixels. Approximately 1 pixel in the TIR image was equal to 4 pixels in the RGB image.

From the presented figures, it is noticeable that there is no universal transformation key for all of the image pairs. Moreover, in all figures, it is possible to read that RMSEx is much lower and stable over all of the distances compared to the RMSEy. RMSEy differs, and there is a certain trend for all cases. The fact that RMSEy is higher could be caused by the relative position between the TIR camera and the RGB camera. The RGB camera is placed around 3 cm above the TIR camera in the same direction, as is Y-axis in the images (vertical direction). In the X-axis direction in the image (horizontal direction), the cameras are, by eye, placed in the same position. Moreover, the optical axis of the cameras is, likely, not ideally parallel, which can make an influence as well.

**Figure 6:**
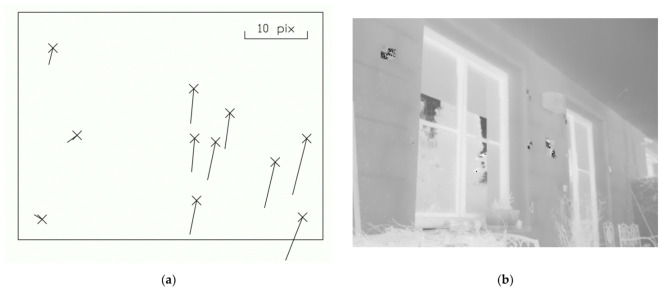
Residuals of the transformed TIR image to the corresponding RGB image using the transformation key computed on image pair 2 m from the façade: (**a**) residuals on the targets (the scale of the residuals were increased); (**b**) thermal infrared image.

The observed trend in RMSEy could be readable. In the case of Figure 5a, the transformation key was computed in the image pair 2 m from the façade. At this distance, the RMSEy is low, 0.4 pixel (on TIR image), and equal to the RMS of the affine transformation. However, when the distance from the test object increases, the RMSEy increases as well. The RMSEy increases more rapidly when the distance is shorter, and when the distance from the façade is longer, the RMSEy increases slower. One could observe the same in the case of the transformation key computed in image pair 11 m from the façade. The lowest RMSEx and RMSEy are at an 11 m distance (Figure 5d). With a decreasing distance, the RMSEy increases, and when the distance is shorter, the RMSEy increases more rapidly. In the case of the transformation key, computed according to the 4 m image pair (Figure 5b) and 6 m image pair (Figure 5c), the RMSEy decreased to the distance where the transformation key was computed and then increased again with the longer distance.

The results showed that it was not possible to find a universal transformation key that could be used for the plane transformation between the TIR and RGB image and the coordinate transformation was dependent on the distance from the object. For close-range photogrammetry, the plane transformation does not seem to be usable. The plane transformation does not particularly seem to be used for very close-range photogrammetry, with distances shorter than 5 m. At close-range photogrammetry, there are also captured images from different convergent angles, and the object structure is very often spatial. Thus, in one image, there could be an object, at the same time, in 2 m distances and 11 m distances. This was tested in one image pair (see Figure 6). The transformation key, which was computed in the 2 m image pair, was used. One could observe that the scene closer to the image (approximately 2 m) shows significantly lower residuals. When the scene is further (approximately 7 m), the residuals rise. All of the main residuals have the same direction.

Nevertheless, the plane transformation could still be used as a proper coordinate transformation. For example, taking the images from the more or less constant distance, especially from a further distance, could still be a reasonable option. One of the scenarios could be capturing the image pairs of the ground with no significant spatial structure (considered a plane, especially from a further distance) from UAV with the same flying altitude during capturing. This should be tested in the following experiments. However, for close-range photogrammetry, the plane transformation is not the right option.

## 3. Spatial Transformation for Reprojection Method

The second suggested method, “Reprojection”, is designed to use a spatial transformation between the RGB image and the corresponding TIR image. The spatial transformation requires knowing the interior orientation of both RGB and TIR cameras and the relative pose between the cameras in the fixed system. At first, the interior orientation was determined by calibration and then the calculated parameters of interior orientation were used for relative pose estimation. The relative pose estimation was carried out by computing the photogrammetric model of the set of image pairs and determining the relative pose between the image pairs.

### 3.1. Camera Calibration

There are two types of calibration of the thermal infrared camera–radiometric calibration and geometric calibration. The radiometric calibration was carried out by a contractor. The geometric camera calibration of the thermal infrared camera is presented for purposes of this article. Via geometric calibration are the estimated parameters of the interior orientation of the camera, which are necessary for the calculations of the suggested method. As the considered parameters of interior orientation, there were camera constant *c*, the position of the principal point *p_x_* and *p_y_*, and coefficients of the radial distortion *k_1_*, *k_2_*. Tangential distortion was not considered, the shear was universally considered as 1, and the scale difference *m* was considered as 0.

The parameters were determined by a camera calibration process using conventional photogrammetry. For the camera calibration, three calibration fields were designed and created.

- 2-level spatial calibration field;
- 3-level spatial calibration field;
- Plane calibration field.

Standard calibration fields are not usable in purposes of calibration of the thermal infrared camera, so calibration fields with visible targets in the TIR image must have been designed.

**Figure 7:**
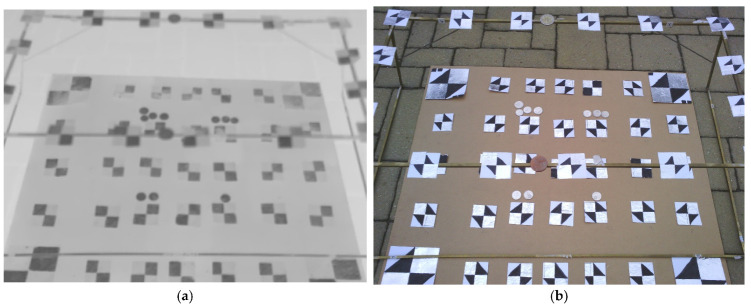
Images for camera calibration using the 2-level calibration field: (**a**) TIR image; (**b**) RGB image.

The 2-level spatial calibration field (Figure 7) was designed according to the suggestions for the spatial calibration field [19]. The calibration field consisted of a plane desk with approximate dimensions (700 × 600 mm) where were placed 35 cross aluminum paper cross targets. A frame was placed on the desk, where another 16 targets were placed. Together, the 2-level spatial calibration field was created. The targets of the calibration field were measured by a total station, from one station, and there were measured distances on the field. The accuracy of the coordinates of the measured targets was determined as 3 mm and the accuracy of the measured distance was determined as 1 mm.

**Figure 8:**
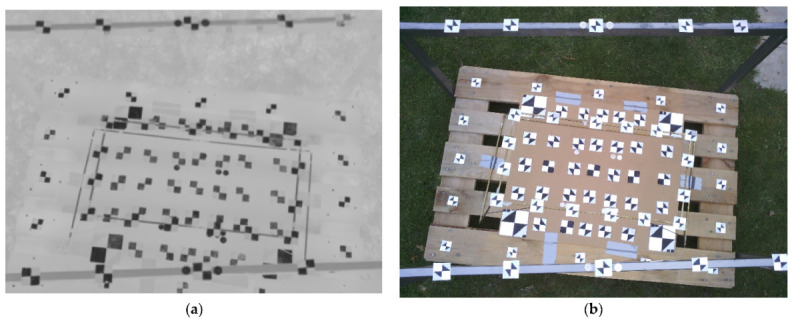
Images for camera calibration using the 3-level calibration field: (**a**) TIR image (**b**) RGB image.

As a second calibration field, a 3-level calibration field was created (see Figure 8), on the same principle as the 2-level calibration field created. The approximate dimension of the 3-level calibration field was 1200 × 800 × 73 mm. The reason for creating the 3-level calibration field was to test if there was improvement in terms of accuracy, and if there was more 3D structure on the calibration field. The base was a plane desk, where the same targets were placed, similar to the 2-level calibration field. Over the desk, a first construction frame was placed, and then over the first construction frame, the second construction frame was placed. On both frames, targets were placed. Targets were measured by total stations and 12 distances were measured by a measuring tape. Both spatial calibration fields were homemade creations. Again, the accuracy of the coordinates of the measured targets was determined as 3 mm and the accuracy of the measured distance was determined as 1 mm.

**Figure 9:**
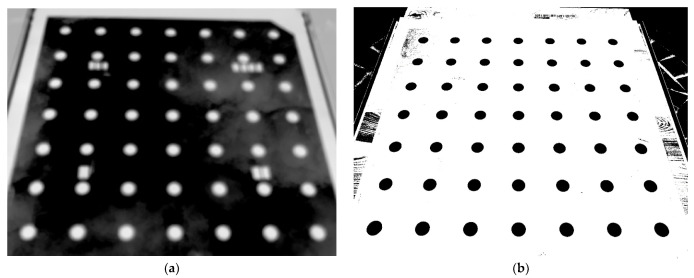
Images for camera calibration using plane calibration field: (**a**) TIR image; (**b**) RGB image.

As a third calibration field, a plane calibration field was created, see Figure 9. In [18], the authors showed that, by using circular targets, it is possible to achieve better results compared to the chessboard plane calibration field. The plane calibration field was created professionally by the manufacturer. The manufacturer advised using stainless steel to have the highest possibility of a firm (not banded) plane field. The calibration field was a 350 × 350 mm desk, 1 mm thick, from stainless steel; in the desk, there were cut-out circles with a diameter of 15 mm. The circles were cut out with a 0.1 mm tolerance of position. The dimensions of the circles were chosen to have enough pixels in the TIR images, if imaging was from a 30 cm distance for the ellipse operator or gravity operator function. The desk was placed on a flat wooden desk. The main idea behind cut-out circles from the calibration field was to have the same effect that we had with cross aluminum paper targets. Stainless steel is a material with low emissivity and the desk was placed on a wooden flat desk, so the holes had high emissivity; then, it was possible to detect the circles on TIR images.

Capturing of images was carried out according to all of the suggested rules by [14,20,21]. On the TIR camera of FLIR E95, it is not possible to change aperture or shutter speed, so it was not possible to operate with focus depth. The TIR camera focused on the 7 m distance using a focusing ring, and the focus was fixed. The influence of changes in the focus to the interior orientation parameters was published in [22]. The RGB camera has a fixed-focus lens. Fixed-focus lenses rely on a very large depth of field. The depth of field of the RGB camera was suspiciously too large. The RGB images were sharp even in very short distances. Theoretically, there is post-processing on RGB cameras to eliminate blurring at such short distances. That could be a potential problem for calibrating the camera on the plane field, where images were captured very close to the plane field (40 cm). However, we had to presume that both cameras had a fixed optical system during the capturing.

For spatial calibration fields, eight images were captured. Four different oblique images with variant intersection angles and four perpendicular images were captured. The roll angle of the camera was rotated by +/−90° to determine the accurate principal point. Each side of the image was fully covered by targets at least once to have the ability to describe the distortion of the camera. In the case of the plane field, pilot experiments showed that eight images are not a sufficient number, probably because of coupling between the parameters of interior orientation and exterior orientation. The experiments showed that adding more convergent images with variant roll angles give more reasonable and stable results. Because of that, the plane field was captured by 16 convergent images with different roll angles and four perpendicular images with different roll angles.

In the case of spatial fields, the targets were measured in images manually. The images were sharp enough to mark targets in the images with 1 pixel accuracy. Targets in images that belonged to the plane calibration field were measured by ellipse or gravity operators. At first, the ellipse operator did not work well in RGB images during the digitization of targets. Thus, the RGB images had to be pre-processed. Contrast of the images had to significantly increase and saturation of the images decrease. This process created more or less binary (black and white) images and it was then possible to use the ellipse operator. The pre-process also solved a problem of a visible thickness (1 mm) of the plane field in convergent images. Spatial calibration fields were large enough to capture the images with a certain distance, where the TIR images focused on the 7 m distances were still sharp. On the other hand, for proper distribution of the points, the plane field had to be captured from a closer distance so the TIR images were noticeably blurred. In the case of blurred circles, it was not possible to use the ellipse operator; the gravity operator was used instead. The different ways of digitizing the points in images led to setting different weights (in the following bundle adjustment process).

**Figure 10:**
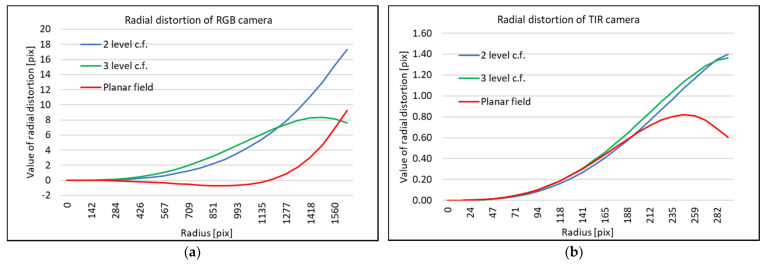
Radial distortion determined by camera calibration using different calibration fields: (**a**) RGB camera (**b**) TIR camera.

The calibration process was computed in the photogrammetric tool Close Range Digital Workstation (CDW). The resulting parameters are presented in Table 4 and Figure 10.

Computed parameters of the interior orientation are very similar in the case of the 2-level calibration field and 3-level calibration field. On the other hand, there is a certain difference between computed principal points based on plane calibration and both multi-level calibration fields.

According to the relatively low values of radial distortion (Figure 10) for both cameras, there is a presumption that the images of both cameras are undistorted by a process in the FLIR E95 camera. Figure 10 shows the highest value of radial distortion around 18 pix in the far image corner; in the case of the TIR camera, the highest value is around 1.4 pix. One could observe (Figure 10a)) different trends of distortion in comparison to the plane calibration field and on both multi-level calibration fields. The difference between the curves is increasing, and in the image width from the center (1296 pix), the difference in the radial distortion value is around 6 pixels. In the case of the TIR camera, the radial distortion is computed on the plane calibration field and both multi-level calibration fields seemed more or less the same from the image center to the image width (232 pix). In the far image corners (232 pix and further from the center), the curves of the radial distance differ by approximately 0.5 pix. In total, absolute values of the radial distortion are low. The resulting radial distortion confirms that the images could be considered undistorted even for plane transformation.

The advantage of the plane and 2-level calibration fields is that both fields are easily portable and can be used for on-site calibration, before and after the capturing images, to check if the optical system of the cameras remained fixed during the capturing.

### 3.2. Estimation of the Relative Pose

The relative pose was determined by a rotation matrix consisting of differences of rotation in three axes Δω, Δφ, and Δκ, and by translation parameters among ΔX, ΔY, and ΔZ. The translation parameters define the position of the TIR camera in the RGB camera coordinate system, when the X-axis is from the optical center of the RGB camera pointing to the right, Y-axis is from the optical center down, and Z-axis is the optical axis of the RGB camera going towards the scene. The parameters of relative orientation define the rotation of the TIR camera in the RGB camera coordinate system. For relative pose determination, all three calibration fields were used again. Each calibration field was captured by eight convergent image pairs in different angles in the fixed configuration using camera FLIR E95. Images were captured in a way to have images covered by targets on the calibration fields, as most as possible. This requirement was complicated by the different fields of view of both cameras. For each calibration field, eight image pairs were processed separately by the conventional photogrammetry process in CDW. For each group of eight images, the interior orientation was used according to the used calibration field. Thus, for example, the resulting interior orientation by calibration, on the plane calibration field, was used also for bundle adjustment when processing the eight image pairs, which also captured the plane calibration field. This is important to note because interior orientation correlates with exterior orientation. According to [23], in particular, principal point estimation from camera calibration strongly correlates with the exterior orientation. The exterior orientations of the images of the eight image pairs were estimated by bundle adjustment. Parameters and results of the bundle adjustment are presented in Table 5. For every image pair, using Equation (4), rotation matrices were computed. The rotation matrices were decomposed to the three angles Δω, Δφ, and Δκ determining the relative rotation between the TIR and RGB camera. Using Equation (5), the relative translation of the cameras was computed. Therefore, for every calibration field, eight relative poses were computed, i.e., eight time parameters ΔX, ΔY, ΔZ, Δω, Δφ, and Δκ. From eight values of each parameter, the mean and its standard deviation were calculated. The mean was considered the final value. The resulting parameters of the relative pose are presented in Table 6. Standard deviations of the means are presented in Table 7.

### 3.3. Verification of Calculated Parameters

**Figure 11:**
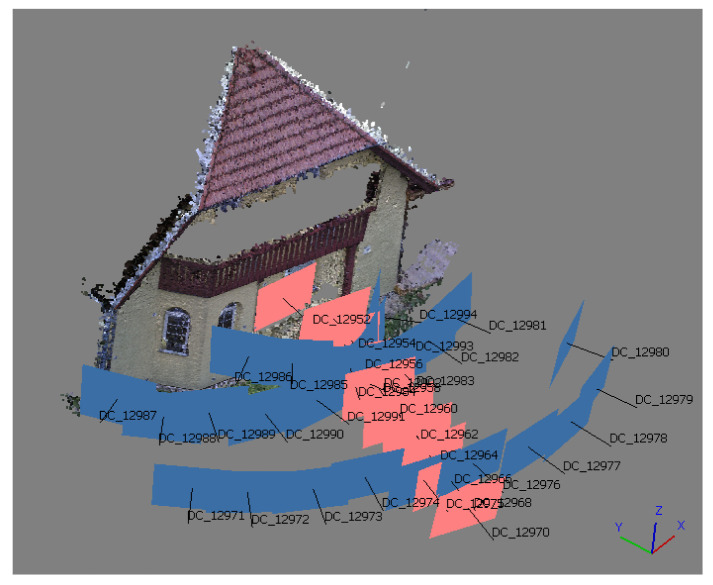
Capturing scenario of the experiment; 10 perpendicular images are highlighted.

An experiment was carried out to test the calculated parameters of the relative pose. A façade of a family house was chosen as a test object for the experiment. Aluminum paper cross targets were placed on the façade. The coordinates of the targets were measured by a total station from the single station. FLIR E95 was used to capture the images in the experiment. For every RGB image, TIR image was also captured. In total, there were 35 image pairs captured. Among the 35 image pairs, there were also 10 image pairs that were captured perpendicular to the façade, at 10 different distances from the façade (2–11 m), the same as it was when testing the plane transformation mentioned in the previous chapter. The sample distances of the image pairs are approximately the same as stated in Table 3. The capturing scenario is presented in Figure 11.

First, only RGB images were processed solely by the SfM method using commercial software Agisoft Metashape. The images were processed in three different projects. For each project, before the processing, there were set parameters of the interior orientation of the cameras according to the used calibration field. All of the inputted values of interior orientation were fixed and were not changed during further processing. After the relative orientation of the images, the geo-referencing on the targets was carried out. The total RMSE of the georeferencing was around 5 mm for all three groups of interior orientation. Parameters of exterior orientation (X, Y, Z, ω, φ, κ) for the 10 perpendicular images were used for testing the computed relative poses.

**Figure 12:**
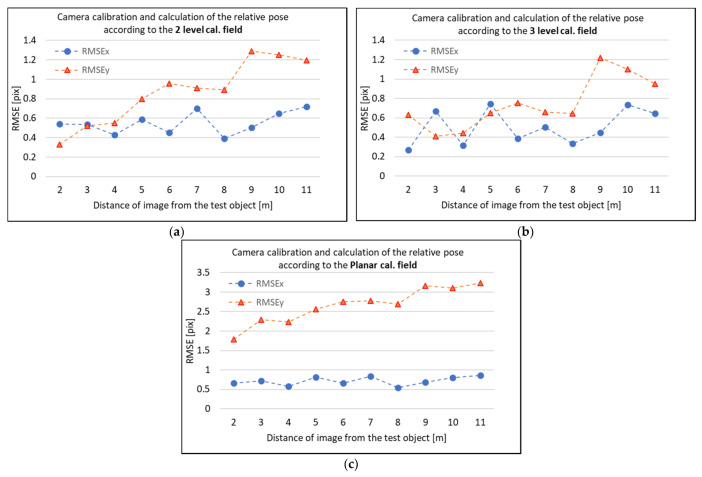
RMSEx and RMSEy of residuals (on TIR images) of spatial transformation using different result parameters ΔX, ΔY, ΔZ, Δω, Δφ, Δκ: (**a**) parameters calculated on the 2-level calibration field; (**b**) parameters calculated on the 3-level calibration field; (**c**) parameters calculated on the plane calibration field.

For each image, of the 10 perpendicular images, the measured photogrammetric targets on the façades were transformed to the RGB camera coordinate system. Then, using the parameters of the relative pose (ΔX, ΔY, ΔZ, Δω, Δφ, Δκ), the points were transformed to the TIR camera coordinate system of the corresponding TIR image. From the TIR camera coordinate system, the points were reprojected to the corresponding TIR image using calculated parameters of the interior orientation. The affiliation to the chosen calibration field was preserved. For example, when a plane calibration field was used for camera calibration and for calculation of parameters of relative poses between the cameras, then the interior orientation was also used for calibration input before processing in Agisoft Metashape. After exterior orientation of RGB images, the relative pose estimated on the plane calibration field was used to transform the point between the camera coordinate systems and, again, the interior orientation calibrated on the plane calibration field was used for reprojection to the TIR image. This was highly important in the case of coupling the parameters of the interior orientation and exterior orientation and relative pose, respectively. The reprojected point to the TIR image was compared to the position of the photogrammetric target in the TIR image, and the residuals were calculated. From the residuals, RMSEx and RMSEy were calculated. The process was repeated for all 10 perpendicular image pairs. The resulting RMSEx and RMSEy are presented in Figure 12. All of the presented RMSE pixel values are related to the TIR image pixel.

According to Figure 12, in the case of the 2-level and 3-level calibration fields, one could observe that the RMSEx and RMSEy are both mostly under the 1-pixel in the corresponding TIR image. The RMSEy is a bit higher, and at some of the distances, RMSEy exceeds 1 pix. It is important to mention that, even though the resulting RMSEs are respectable, the TIR image has significantly low-resolution compared to the RGB image, and even 1 pixel does matter.

RMSEy can be influenced by the Y coordinate of the principal point of the TIR camera and also by the Y coordinate of the principal point of the RGB camera (which influences the exterior orientation angle ω_RGB_). RMSEy is also influenced slightly by parameter ΔY of the relative pose and by Δω. On one TIR image, all residuals were more or less constant with the same direction. The testing showed that estimation of the translation between the cameras does not have such influence, but the estimation of the relative rotation influences the result strongly. The difference of 0.05° in Δω can cause the difference of 0.5 pix in the TIR image. According to Table 7, Δω was calculated with σ_Δ_ω 0.03°–0.06°. Estimation of the Δω angle can then have a high influence. There are insufficient results in the case of the plane calibration field (Figure 12c). However, the result is still better compared to the plane transformation and does not differ with too many overall distances (1.8 pix for 2 m and 3. 2 pix for 11 m). From Figure 12c, it is (slightly) possible to observe a trend in the case of the plane calibration field, but there is not enough data to make a proper statement. In the case of the plane calibration field, insufficient results may have their core in the insufficient interior orientation determination, as was previously discussed.

## 4. Conclusions

To obtain accurate 2D and 3D models, enriched with information derived from the original thermal infrared image, it seems necessary to come up with certain methods of photogrammetric co-processing of RGB and TIR images. Two of the methods were previously suggested by the authors and they are also briefly presented in this article. Both methods are designed for close-range photogrammetry purposes. For the first method, Sharpening, the plane transformation was designed. For the second method, the spatial transformation was designed. Determining and testing the transformations were the main goals of this article. For purposes of this paper, a handheld TIR camera, FLIR E95, equipped by an RGB camera, was used. Cameras like this offer, naturally, a fixed system of TIR and RGB cameras. This advantage could be used for photogrammetric co-processing.

For plane transformation (affine transformation, in our case), it was necessary to use undistorted images captured by cameras relatively close to each other, with the same direction of their optical axes. Then the transformation key for the affine transformation was computed. An experiment was carried out to see if there was a possibility of finding a universal transformation key, which could be used for all other image pairs of the dataset. The universal transformation key would let us avoid the process of computing a unique transformation key for every image pair, which would make the Sharpening method difficult and significantly less automatic. However, the experiment showed that, in the case of our dataset, it was not possible to find a universal transformation key. The resulting RMSEy was dependent on the distance of the object to the image. This was probably caused by the shift of the TIR and RGB camera (the RGB camera was physically placed 3 cm above the TIR camera). According to the results of the experiment, there is potential to use the plane transformation for purposes of photogrammetry, where there are longer distances to the object, and where the distance of the capturing is more or less constant. One of the applications could be to use UAVs from constant mid-range distances, capturing the ground where surface changes are not significant. However, the article is focused on close-range distances, and at close-range photogrammetry, there are expected substantial differences in the distance on the scene during the capturing. For close-range photogrammetry, plane transformation does not seem sufficient.

Spatial transformation is designed for the Reprojection method. Spatial transformation also requires a fixed system of TIR and RGB cameras. The advantage of spatial transformation is that the process does not require undistorted images. Spatial transformation requires geometric calibration of both cameras and estimation of the relative pose between TIR and RGB cameras. The relative pose consists of relative translation with parameters ΔX, ΔY, ΔZ, and relative rotation with parameters Δω, Δφ, and Δκ. The process of geometric calibration and estimation of the relative pose is described in this paper. The experiments showed that, during camera calibration, it is very important to decouple parameters of the interior and exterior orientation. For estimation of the relative pose, it is important to use decoupled interior orientation parameters, and accuracy, and precisely determine the exterior orientation of the images, of the image pairs. This requires a short imaging distance (larger sample distance), proper intersection angles, and accurate control points on the scene. In the provided experiments, the relative pose was estimated on three different calibration fields. The best results showed estimation on the 3-level calibration field. The standard deviation of the mean of the calculated translation parameter was less than 1 mm and the rotation parameter less than 0.04°. The calculated parameters were tested on 10 different image pairs with all of them captured perpendicular to the façade. The exterior orientation of 10 RGB images of the image pair was determined by the SfM method when processed together with several other images of the façade. The testing showed that the distance does not have a major influence on the resulting RMSE in the TIR image. RMS was oscillating around 0.5 pix and RMSEy was a bit higher, 0.8 pix, but, twice, RMSEy little exceeded the 1 pixel value. RMSE 1 pixel is still respectable, but a pixel in the TIR image has a larger sampling distance due to lower resolution of the TIR image compared to the RGB image.

Overall, the testing of spatial transformation showed better reliability. For close-range purposes, it is not sufficient to use plane transformation and it is advisable to use spatial transformation instead. If the spatial transformation was also used for the Sharpening method, the method would be an alternative to the Reprojection method. Based on the conclusions of this article, the Sharpening method must be redesigned and the spatial transformation must be used. The authors believe that the way on “how” will be via spatial transformation implementation being original. Detailed descriptions of processes, of both methods and results of photogrammetric co-processing using those methods, will be presented in a following article.

## Figures and Tables

**Table 1:** Comparison of parameters of handheld thermal infrared cameras [15,16].

Camera	TIR Camera Resolution (pix)	RGB Camera Resolution (pix)
FLIR E75	320 × 240	5 MP
FLIR E85	384 × 288	5 MP
FLIR E95	464 × 348	5 MP
FLIR E96	640 × 480	5 MP
Fluke TiS60+	320 × 240	5 MP
Fluke Ti480 PRO	640 × 480	5 MP
Fluke TiX501	640 × 480	5 MP

**Table 2:** Parameters of TIR camera and RGB camera of FLIR E95.

Parameter	TIR Camera	RGB Camera
Focal length (mm)	10	3.29
Sensor size (mm)	7.89 × 5.92	3.67 × 2.74
Resolution (pix)	464 × 348 pix	2592 × 1944
Size of a pixel (mm)	0.017 mm	0.0014 mm
FOV (°)	42° × 32°	53° × 41°

**Table 3:** Approximate sample distances for images captured from different distances.

Distance from the Object		2 m	3 m	4 m	5 m	6 m	7 m	8 m	9 m	10 m	11 m
**Sample distance (mm)**	**TIR** **image**	3.4	5.1	6.8	8.5	10.2	11.9	13.6	15.3	17.0	18.7
	**RGB** **image**	0.9	1.3	1.7	2.2	2.6	3.0	3.4	3.9	4.3	4.7

**Table 4:** The resulting interior orientations of the TIR camera and the RGB camera of FLIR E95, determined on different calibration fields.

		c (pix)	Px (pix)	Py (pix)
TIR camera	2-level calibration field	592.8	−2.7	1.0
	3-level calibration field	593.5	−3.3	1.4
	Plane calibration field	591.8	−2.6	2.1
RGB camera	2-level calibration field	2483.5	−26.5	25.3
	3-level calibration field	2481.4	−23.4	27.1
	Plane calibration field	2472.3	−29.1	31.8

**Table 5:** Parameters and results of the bundle adjustment. (The software that was used, where the bundle adjustment was computed, uses different angle combinations: azimuth, tilt, swing).

		Distance from the Object (m)	Sample Distance (mm)	Avg. Residuum on Image Coordinates X	Avg. Residuum on Image Coordinates Y	σX (mm)	σY (mm)	σZ (mm)	σα (mm)	σν (mm)	σκ (mm)
**2-level calibration field**	TIR	0.9	1.5	0.28	0.29	0.89	0.67	0.95	0.057	0.060	0.046
	RGB	0.9	0.4	0.96	1.11	0.90	0.70	0.95	0.058	0.063	0.047
**3-level calibration field**	TIR	1.5	2.6	0.24	0.23	1.13	0.82	1.27	0.048	0.049	0.040
	RGB	1.5	0.6	0.88	1.00	1.12	0.82	1.26	0.048	0.050	0.040
**Plane calibration field**	TIR	0.4	0.7	0.13	0.24	0.23	0.26	0.26	0.036	0.062	0.061
	RGB	0.4	0.2	0.72	0.91	0.33	0.31	0.32	0.047	0.078	0.078

**Table 6:** The resulting parameters ΔX, ΔY, ΔZ, Δω, Δφ, and Δκ.

	ΔX (m)	ΔY (m)	ΔZ (m)	Δω (°)	Δφ (°)	Δκ (°)
**2 level calibration field**	−0.0001	−0.0246	−0.0045	−0.800	0.078	−0.009
**3 level calibration field**	−0.0002	−0.0248	−0.0065	−0.833	−0.061	−0.007
**Plane calibration field**	−0.0002	−0.0252	−0.0061	−0.663	0.163	−0.020

**Table 7:** The standard deviation of the mean of parameters ΔX, ΔY, ΔZ, Δω, Δφ, Δκ.

	σ_ΔX_ (m)	σ_ΔY_ (m)	σ_ΔZ_ (m)	σ_Δ_ω (°)	σ_Δ_φ (°)	σ_Δ_κ (°)
**2 level calibration field**	0.0008	0.0005	0.0004	0.031	0.047	0.027
**3 level calibration field**	0.0007	0.0008	0.0004	0.036	0.029	0.016
**Plane calibration field**	0.0006	0.0005	0.0001	0.058	0.065	0.041
